# Pectoralis major radiation recall

**DOI:** 10.1002/jmrs.303

**Published:** 2018-09-09

**Authors:** Emma Hack, Thanuja Thachil, Narayan Karanth

**Affiliations:** ^1^ Department of Medicine Royal Darwin Hospital Tiwi Northern Territory Australia

**Keywords:** adverse events, chest, clinical site, discipline, general, medical imaging, radiation oncology

## Abstract

Radiation recall is an uncommon phenomenon describing an acute localised inflammatory toxicity affecting tissue previously exposed to radiotherapy. It is precipitated by administration of certain medications, including chemotherapy. We describe a case involving a 50‐year‐old Aboriginal male smoker from a remote community in Northern Australia who underwent treatment for stage IV non‐small cell lung cancer with localised radiotherapy to the primary right upper lung lobe tumour. This was followed by a course of gemcitabine, which was ceased prematurely after four cycles when he presented with radiation recall to his right pectoralis major. Our case description is complemented with a brief review of current literature regarding our case and gemcitabine‐related radiation recall. This was in the context of concurrent musculoskeletal strain, an as‐yet unreported association with radiation recall. His condition settled with steroid administration and discontinuation of gemcitabine.

## Case Report

A 50‐year‐old Aboriginal male smoker from a remote community in Northern Australia presented with a 6‐month history of weight loss and anaemia. Subsequent investigation revealed a 62 × 111 × 72 mm stage IV right upper lobe non‐small cell lung adenocarcinoma (epidermal growth factor receptor, anaplastic lymphoma kinase, kirsten rat sarcoma viral oncogene mutation wild‐type) with supraclavicular nodal and splenic metastases (T_3_N_3_M_1b_). Other medical history included latent tuberculosis for which he was taking isoniazid 250 mg daily and pyridoxine 25 mg daily, chronic kidney disease, emphysema managed with salbutamol inhaler as needed and hypertension treated with ramipril 1.25 mg daily.

**Figure 1:**
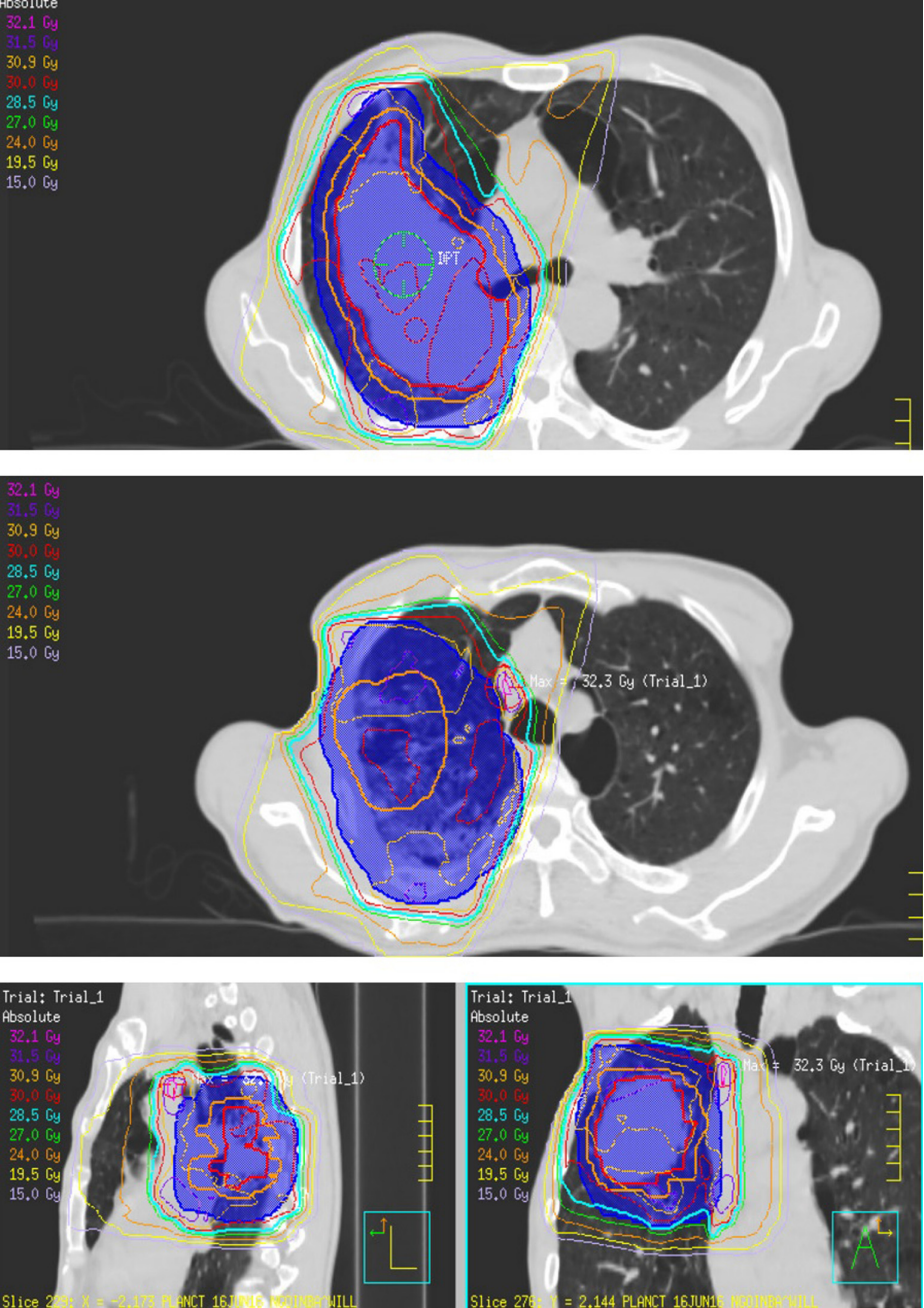
Radiotherapy planning. Pectoralis major received 15–30 Gy. Gross tumour volume = red colour wash, clinical tumour volume = orange colour wash, planned tumour volume = blue colour wash.

**Figure 2:**
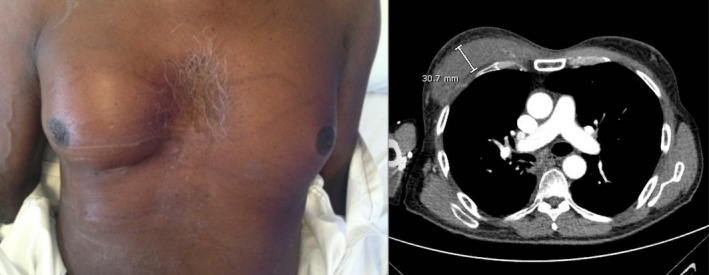
Right pectoralis major swelling.

He relocated to a tertiary medical facility to undergo palliative chemoradiotherapy. He completed 2 weeks of radiotherapy with four beams at 20–30 Gy in 10 fractions with 3D conformal technique to the primary tumour, with planning target volume covered by 95% of the isodose line. The ipsilateral breast including pectoralis major received dose ranging from 15 to 30 Gy (Fig. 1). One month later, he commenced three weekly cycles of palliative chemotherapy with gemcitabine and carboplatin. One week after his fourth cycle he presented to the local emergency department with increasing pain and swelling to the right breast (Fig. 2). He had participated in heavy lifting 2 weeks prior and recalled bilateral aching to his arms following the activity. He had not commenced any other medications and did not drink alcohol.

**Figure 3:**
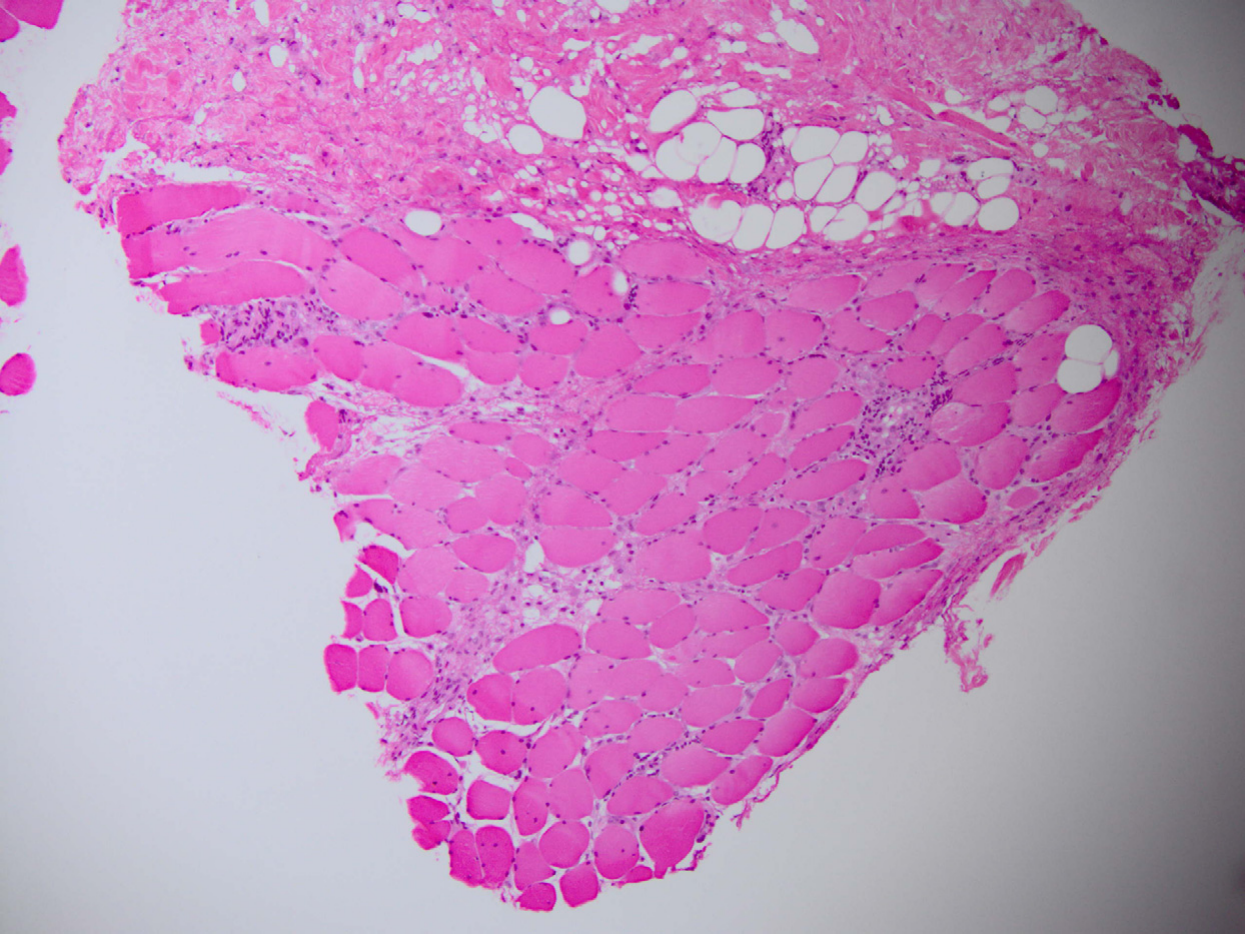
Muscle biopsy showed non‐specific myositis.

The patient was haemodynamically stable and afebrile. Marked right breast asymmetry was noted with a firm, immobile, tender, warm right breast swelling. There were no overlying skin changes. Laboratory results revealed an acute kidney injury with creatinine level 123 μmol/L (60–110) and egfr 52 mL/min/1.72m^2^ (baseline 65–70) along with an elevated creatine kinase at 374 IU/L (40–200). White cell count was normal, though C‐reactive peptide was elevated at 94 mg/L (<5). Haemoglobin was 93 g/L unchanged from previous (130–180). Anti‐signal recognition antibodies were positive. Transcription intermediary factor 1‐gamma antibodies were negative. Incision and exploration of the swelling showed diffuse muscular hypertrophy with no evidence of abscess or haematoma. Subsequent biopsy confirmed acute non‐specific myositis (Fig. 3). Microscopy and culture were negative. The patient gave permission for the case report to be published.

## Discussion

Radiation recall is an acute localised inflammatory toxicity affecting tissue previously exposed to radiotherapy. It is always precipitated by administration of specific medications. Since the phenomenon was first described in literature in 1959,^1^ it has been associated with a number of agents including chemotherapy,^2^, ^3^ targeted therapies (such as mammalian target of rapamycin or epidermal growth factor receptor inhibitors),^4^ antibiotics,^5^, ^6^, ^7^ anti‐oestrogen therapy^8^ and statins.^9^, ^10^ In general, manifestation of radiation recall varies, and may range from mild superficial cutaneous reactions such as dermatitis, to less‐common deeper reactions including myositis.^11^, ^12^ Pathogenesis is unclear though it is hypothesised that radiation recall represents a variant of idiosyncratic hypersensitivity as accumulative previous cell damage from radiotherapy sensitises tissue to the cytotoxic effects of certain drugs.^5^

Chemotherapeutic agents are particularly linked to the phenomenon which can occur at any time varying from days to years after completing radiotherapy. Gemcitabine is an antimetabolite chemotherapeutic agent frequently used as first line treatment in non‐small cell lung cancer. Gemcitabine‐related radiation recall has been implicated in a number of case reports, manifesting as dermatitis,^13^, ^14^, ^15^ myositis,^13^, ^16^, ^17^, ^18^, ^19^ dermatomyositis,^20^ pericardial effusion,^21^ pulmonary fibrosis,^22^ vaginal necrosis,^23^ rectal haemorrhage,^24^ gastritis,^25^ esophagitis,^2^ and compartment syndrome.^26^ It has also been suggested that as compared to other agents, gemcitabine‐associated radiation recall may be more frequently associated with deeper‐organ and tissue reactions (such as myositis) as opposed to superficial reactions such as dermatitis.^7^ Furthermore, there are reports of a dose‐responsive relationship between amount of radiotherapy received and risk of developing radiation recall.^27^

There are few case reports specifically documenting gemcitabine‐associated radiation recall of pectoralis major – though we identified two cases in patients receiving treatment for non‐small cell lung cancer^28^, ^29^ and one in a patient with non‐hodgkins lymphoma.^30^ Musculoskeletal injury as a precipitant or contributing factor to radiation recall has not been reported in the literature to our knowledge.

There are no widely accepted guidelines on managing radiation recall. The consensus in available literature indicates discontinuation of the chemotherapeutic agent coupled with steroid or anti‐inflammatory treatment seems to resolve the condition. Many clinicians recommend permanent discontinuation of the causative medication while others suggest re‐challenging the patient with the offending chemotherapeutic agent with some reports of success.^29^, ^31^

Our patient commenced a weaning course of oral dexamethasone and the chemotherapy regime was discontinued with clinical improvement over several weeks. Future plans for administration of chemotherapeutic agents for this patient were abandoned. He later commenced a programmed cell death protein 1 inhibitor in the form of nivolumab as second‐line therapy.

Our patient experienced localised pectoralis major radiation recall, possibly unique as a result of gemcitabine potentiated by concurrent musculoskeletal strain in the context of radiotherapy given 4 months earlier. Radiation recall has important clinical implications for patients with cancer and may affect the clinician's ability to administer important treatment.

## Conflict of Interest

The authors declare no conflict of interest.
